# Digital Cognitive Behavioral Therapy for Insomnia Versus Sleep Hygiene Education for Prevention of Perinatal Depression: Protocol for a Randomized Clinical Trial

**DOI:** 10.2196/90969

**Published:** 2026-05-26

**Authors:** Jennifer N Felder, Patricia J Moran, Bernadette McClelland, Richelle Mah, Candance Sorensen, Carolyn Ponting, Nasim C Sobhani, John Neuhaus, Andrew Krystal, Rachel Manber

**Affiliations:** 1 Osher Center for Integrative Health, University of California, San Francisco, UCSF Box 1726, San Francisco, CA, 94143, United States, 1 415-476-7014; 2 Department of Psychiatry and Behavioral Sciences, University of California, San Francisco, San Francisco, CA, United States; 3 Department of Counseling Psychology and Human Services, University of Oregon, Eugene, OR, United States; 4 Department of Obstetrics, Gynecology, and Reproductive Sciences, University of California, San Francisco, San Francisco, CA, United States; 5 Department of Epidemiology and Biostatistics, University of California, San Francisco, San Francisco, CA, United States; 6 Department of Psychiatry and Behavioral Science, Stanford University, Palo Alto, CA, United States

**Keywords:** insomnia, depression, prevention, perinatal, pregnant, postpartum, cognitive behavioral therapy, sleep hygiene education, randomized controlled trial, study protocol

## Abstract

**Background:**

Insomnia during pregnancy is a modifiable risk factor for depression. Research in nonpregnant populations and preliminary findings in a pregnant population suggest that targeting insomnia with digital cognitive behavioral therapy for insomnia (CBT-I) may prevent depression.

**Objective:**

This study aims to evaluate the efficacy of digital CBT-I for the prevention of depression through 12 months post partum compared to that of a credible, clinically relevant comparator intervention, sleep hygiene education (SHE). This project will also provide valuable information about how and for whom CBT-I prevents perinatal depression.

**Methods:**

The Perinatal Research for Improving Sleep and Mental Health study is a fully remote, 2-arm parallel-group randomized controlled trial designed to test the superiority of digital CBT-I versus SHE for the prevention of perinatal depression. We aimed to randomize a target sample of at least 443 pregnant participants with insomnia disorder to digital CBT-I or SHE at a 1:1 ratio. Participants randomized to digital CBT-I received SleepioRx (Big Health, Inc), which uses sleep consolidation, stimulus control, cognitive therapy, relaxation techniques, and SHE in 6 interactive modules (5‐15 min each). Participants randomized to SHE received information about sleep hygiene, the impact of sleep on performance, healthy sleep habits, lifestyle influences on sleep, and creating a sleep-friendly bedroom in 6 weekly digital handouts. Data are collected at baseline, 5 weeks after randomization, 10 weeks after randomization, 36 weeks’ gestation, and 3, 6, 9, and 12 months post partum. The primary outcome is depression incidence, measured using the Structured Clinical Interview for *DSM-5* (*Diagnostic and Statistical Manual of Mental Disorders* [Fifth Edition]) Disorders, Research Version. Other outcome measures include the Edinburgh Postnatal Depression Scale, Patient Health Questionnaire-9, Columbia Suicide Severity Rating Scale, Generalized Anxiety Disorder Scale, and a birth outcomes questionnaire. Mediator measures include the Insomnia Severity Index, Consensus Sleep Diary, Structured Clinical Interview for Sleep Disorders, Perseverative Thinking Questionnaire, Dysfunctional Beliefs about Sleep Scale, Pre-sleep Arousal Scale, and Difficulties in Emotion Regulation-Short Form.

**Results:**

Data collection began in November 2022 and is projected to end in December 2026.

**Conclusions:**

If study hypotheses are supported, then this project will provide a new, scalable approach for preventing perinatal depression.

## Introduction

Perinatal depression is a prevalent problem, experienced by 1 in 7 pregnant or postpartum individuals [1]. It is associated with long-lasting consequences, including increased risk of suicide [2], impairments in parenting [3], and immense societal costs [4]. Accordingly, there is a significant body of research investigating interventions to prevent perinatal depression [5]. The majority of these intervention trials focused on individuals with elevated depressive symptoms or a previous history of depression. In contrast, treating insomnia during pregnancy represents a promising but underexplored avenue for reducing perinatal depression risk.

As is true for nonpregnant individuals [6], insomnia during pregnancy is associated with increased risk for depression [7-9]. Numerous randomized controlled trials (RCTs) have shown that insomnia during pregnancy is modifiable with cognitive behavioral therapy for insomnia (CBT-I), whether offered face-to-face or via digital app [10-12]. Furthermore, findings from a post hoc analysis suggest that treating insomnia during pregnancy may prevent depression. Nondepressed pregnant individuals randomized to digital CBT-I were less likely to report elevated depression symptoms at 3 months post partum relative to those randomized to usual care (3/86, 4% vs 15/85, 18%; *P*=.006) [13]. These findings are consistent with previous research among nonpregnant individuals, indicating that targeting insomnia with digital CBT-I halved the incidence of moderate-to-severe depression relative to an attentional control [14].

Based on these promising post hoc findings in a perinatal sample and relevant findings in nonpregnant populations, we are conducting a confirmatory efficacy trial that is sufficiently powered and designed to provide definitive evidence about whether treating prenatal insomnia prevents perinatal depression. We are conducting a masked RCT to evaluate the efficacy of digital CBT-I for the prevention of depression through 12 months post partum compared with a credible, clinically relevant comparator group, sleep hygiene education (SHE). We hypothesize that participants randomized to digital CBT-I will have significantly lower rates of incident major depression during the perinatal period (hypothesis 1a) and lower depressive symptoms from baseline to 12 months post partum (hypothesis 1b) relative to participants randomized to digital SHE.

This project will also provide valuable information about *how* and *for whom* CBT-I prevents perinatal depression. Based on our preliminary data, we hypothesize that a change in prenatal insomnia symptom severity will mediate the effect of digital CBT-I on perinatal depression incidence (hypothesis 2a) and perinatal depressive symptom severity (hypothesis 2b). Secondary mediators include perseverative negative thinking, dysfunctional beliefs about sleep, nocturnal cognitive arousal, and emotion regulation. Finally, we hypothesize that baseline depressive symptom severity will moderate the effect of digital CBT-I on perinatal depression incidence (hypothesis 3a) and depressive symptom severity from baseline to 12 months post partum (hypothesis 3b).

## Methods

### Trial Design

The Perinatal Research for Improving Sleep and Mental Health (PRISM) study is a fully remote, 2-arm parallel-group RCT designed to test the superiority of digital CBT-I versus SHE. We completed enrollment for the PRISM study in June 2025 and expect to complete data collection in December 2026.

### Eligibility Criteria

We selected eligibility criteria to include participants who are appropriate for an intervention targeting insomnia during pregnancy to prevent depression, and to exclude participants for whom theory or empirical data suggest digital CBT-I is likely to be ineffective, or for whom the effect of the intervention would be difficult to interpret. Full eligibility criteria, rationale, and ascertainment are presented in Table 1.

**Table 1.** Eligibility criteria, rationale, and ascertainment.

Criteria	Rationale	Ascertainment
Inclusion criteria		
Pregnant 14‐28 weeks gestation	Women who experience a miscarriage will become ineligible and will be withdrawn. We chose the lower limit of 14 weeks because the rate of first trimester miscarriage in women with a missed menstrual period and positive urine pregnancy test is 12%‐24% [15], and the upper limit of 28 weeks so women have time to complete the interventions before birth.	Self-report; documentation of pregnancy (eg, dated ultrasound image and after visit summary)
18 years or older	Adolescents have unique sleep needs.	Self-report
Daily access to a web-enabled computer, smartphone, or tablet	Interventions will be delivered digitally, and the sleep diary requires daily monitoring of sleep via online questionnaires. In 2019, more than 90% of adults aged 18‐49 years had a smartphone and 77% had broadband internet at home; 91% of women 18‐49 years old with low income had a smartphone or high-speed broadband internet at home, thus mitigating concerns that requiring daily internet access will exclude individuals with low income [16].	Self-report
Current elevated insomnia symptom severity and insomnia disorder	We will include women who are likely to meet criteria for insomnia disorder using validated cutoffs on the ISI^a^ [17]. Insomnia disorder is an inclusion criterion because CBT-I^b^ is indicated for insomnia disorder [18]. At screening, we excluded women unlikely to meet criteria for insomnia disorder using a cutoff validated for identifying participants in clinical trials (ISI<11) [17]. This cutoff yielded 97% sensitivity and 100% specificity when compared with a semistructured diagnostic interview [17]. At baseline, we included those with elevated insomnia symptom severity, defined as a cutoff ≥10 [17,19].	ISI≥11 at screening; SCISD^c^ [20]; ISI≥10 at baseline
English speaking	The digital CBT-I app is currently available in English only.	Self-report
Exclusion criteria		
Current major depression	Our focus is on preventing, not treating, perinatal depression.	SCID-5-RV^d^ [21]
Taking or planning to take ADM^e^	ADM use would make the effect of CBT-I on preventing depression difficult to interpret. This includes medications that work as an antidepressant or may have antidepressant effects (ie, Lamictal) as well as antidepressant treatments (ie, TMS^f^).	Self-report
Other diagnosed or suspected sleep disorder	We will exclude sleep disorders not likely to benefit from digital CBT-I (ie, hypersomnolence disorder, circadian rhythm sleep-wake disorder [delayed, advanced, shift work, irregular, non-24, and unspecified], obstructive sleep apnea, restless legs syndrome, nightmare disorder, non-REM^g^ sleep walking, non-REM sleep terror, REM sleep behavior disorder, and narcolepsy).	SCISD; validated screeners for prenatal sleep apnea [22]
Other psychiatric or medical issues (eg, bipolar disorder, active suicidality, bed rest, and epilepsy)	We will exclude people who have any psychiatric or medical issues that necessitate priority treatment, could be exacerbated by the sleep restriction component of CBT-I, or would interfere with participation.	SCID-5-RV; QuickSCID^h^; Columbia-Suicide Severity Rating Scale; Self-report
Night shift worker	CBT-I would likely be ineffective, or the effect would be difficult to interpret.	Self-report

a ISI: Insomnia Severity Index.

b CBT-I: cognitive behavioral therapy for insomnia.

c SCISD: Structured Clinical Interview for Sleep Disorders, Revised.

d SCID-5-RV: Structured Clinical Interview for *DSM-5* (*Diagnostic and Statistical Manual of Mental Disorders* [Fifth Edition]) Disorders, Research Version.

e ADM: antidepressant medication.

f TMS: transcranial magnetic stimulation.

g REM: rapid eye movement.

h QuickSCID: Quick Structured Clinical Interview for *DSM-5* Disorders.

### Intervention and Comparator

#### Digital CBT-I

Digital CBT-I was delivered using SleepioRx (Big Health, Inc). SleepioRx was cleared by the US Food and Drug Administration for the treatment of insomnia in August 2024 and was formerly known as Sleepio. SleepioRx delivers CBT-I and includes sleep consolidation, stimulus control, cognitive therapy, relaxation techniques, and SHE. Content is delivered in 6 engaging levels (5‐15 min each). The program is interactive, automated, and tailored to participant progress. Participants receive reminders to complete each level. In March 2025, SleepioRx was replatformed with the new app, only accessible via smartphone and tablet applications (iOS [Apple Inc] and Android [Google]), whereas the previous version was also available via a web app. All therapeutic content, techniques, and sequencing were unchanged.

Engagement is monitored by the study team. Each week, Big Health, Inc sent a file detailing each participant’s use of SleepioRx (eg, session start and end time and number of sleep diaries completed). Unmasked study staff members uploaded this file to REDCap (Research Electronic Data Capture) to monitor adherence to intervention protocols. Unmasked staff members contacted participants who were overdue ≥4 days on a session to identify and troubleshoot barriers.

Additionally, participants in this arm received supplemental content about prenatal, postpartum, and infant sleep that was previously evaluated in a trial of therapist-delivered CBT-I for prenatal insomnia [11]. This content was delivered via 3 separate emails at randomization, 36 weeks gestation, and 3 months post partum, and included sleep hygiene recommendations specific for pregnancy, suggestions for improving postpartum sleep, instructions on how to adjust the time in bed prescription during the postpartum period, psychoeducation about infant sleep, and tips for promoting healthy sleep development among babies 0‐3 months and 3‐6 months of age.

#### Digital SHE

Our research question is whether digital CBT-I outperforms a clinically relevant comparator. SHE has face validity, is frequently used in clinical practice [23,24], and is rated as credible as CBT-I [25]. SHE matches the CBT-I intervention in delivery format (digital), frequency (weekly), and number of sessions (n=6). SHE was delivered via link to a REDCap survey page. It includes information about sleep hygiene, the impact of sleep on performance, healthy sleep habits, lifestyle influences on sleep, and creating a sleep-friendly bedroom. At the end of each session, participants were asked: “What key points from today’s session do you want to keep in mind this week?” They received via email a PDF of the handout and their response after submitting the questionnaire. Study staff members did not monitor SHE adherence. As a result, participants in the digital CBT-I condition had the opportunity for increased program support and staff contact.

#### Concomitant Care

Concomitant nonstudy treatment for insomnia or depression is not prohibited; use of nonstudy treatments is tracked in a nonstudy treatment questionnaire at each assessment time point.

### Outcomes

#### Overview

Table 2 displays all primary, secondary, and exploratory measures, time points, and their allowable response windows. Per SPIRIT (Standard Protocol Items: Recommendations for Interventional Trials) 2025 guidelines [26], we focus the following description on primary and secondary outcomes.

**Table 2.** Primary, secondary, and exploratory measures, time points, and allowable response windows.

Allowable window^a^	Screening survey (up to 5 weeks before randomization)	Screening interview (up to 1 month before randomization)	Baseline (up to 2 weeks before randomization)	5 week post randomization (+/− 7 days)	10 week post randomization (+/− 7 days)	36 week gestation (+/− 7 days)	3 months post partum (+/− 1 month)	6 months post partum (+/− 1 month)	9 months post partum (+/− 1 month)	12 months post partum (+/− 1 month)
Primary clinical outcome measures										
SCID-5-RV^b^ (primary)		✓			✓	✓	✓	✓	✓	✓
EPDS^c^			✓	✓	✓	✓	✓	✓	✓	✓
PHQ-9^d^			✓	✓	✓	✓	✓	✓	✓	✓
Secondary clinical outcome measures										
C-SSRS^e^		✓			✓	✓	✓	✓	✓	✓
GAD-7^f^			✓		✓	✓	✓	✓	✓	✓
Birth outcomes							✓			
Primary mediator measures										
ISI^g^ (primary)	✓	✓		✓	✓	✓	✓	✓	✓	✓
SCISD^h^		✓			✓	✓	✓	✓	✓	✓
Sleep diary	✓				✓					
PSQI^i^			✓		✓	✓	✓	✓	✓	✓
Secondary mediator measures										
PTQ^j^			✓		✓	✓	✓	✓	✓	✓
DBAS-16^k^			✓		✓	✓	✓	✓	✓	✓
PSAS^l^			✓		✓	✓	✓	✓	✓	✓
DERS-SF^m^			✓		✓	✓	✓	✓	✓	✓
Maternal-infant outcomes										
BISQ-R^n^							✓	✓	✓	✓
IBQ-R^o^							✓	✓	✓	✓
MPAS^p^							✓	✓	✓	✓
Miscellaneous measures										
Demographics	✓									
Documentation of pregnancy	✓									
TEQ^q^^r^										
Birth date questionnaire^s^										
Nonstudy treatment questionnaire			✓		✓	✓	✓	✓	✓	✓
Adverse events interview					✓	✓	✓	✓	✓	✓
CSQ^t^					✓					✓
Exit questionnaire					✓					✓
WHODAS 2.0^u^			✓							
*DSM-5*^v^ crosscutting measure			✓							
PRAMS SLE-14^w^							✓			
QuickSCID^x^		✓								
TFA^y^^z^										

a For the 5 weeks post randomization, 10 weeks post randomization, and 36 weeks gestation time points, the preferred response window is +/− 7 days, but we will accept +/− 14 days in exceptional circumstances. The 36 weeks gestation time point will not be collected if <7 days from the 10 weeks post randomization timepoint. If we are unable to obtain information about the date the participant delivered her baby, the postpartum time points will be tied to her estimated due date that is provided at screening.

b SCID-5-RV: Structured Clinical Interview for *DSM-5* (*Diagnostic and Statistical Manual of Mental Disorders* [Fifth Edition]) Disorders, Research Version.

c EPDS: Edinburgh Postnatal Depression Scale.

d PHQ-9: Patient Health Questionnaire.

e C-SSRS: Columbia Suicide Severity Rating Scale.

f GAD-7: Generalized Anxiety Disorder Scale.

g ISI: Insomnia Severity Index.

h SCISD: Structured Clinical Interview for Sleep Disorders, Revised.

i PSQI: Pittsburgh Sleep Quality Index.

j PTQ: Perseverative Thinking Questionnaire.

k DBAS-16: Dysfunctional Beliefs About Sleep scale.

l PSAS: Pre-sleep Arousal Scale.

m DERS-SF: Difficulties in Emotion Regulation Scale-Short Form.

n BISQ-R: Brief Infant Sleep Questionnaire – Revised.

o IBQ-R: Infant Behavior Questionnaire – Revised.

p MPAS: Maternal Postnatal Attachment Scale.

q TEQ: Treatment Credibility and Expectancy Questionnaire.

r TEQ will be completed at randomization.

s Birth date questionnaire will be completed 3 weeks after a participant’s expected due date.

t CSQ: Client Satisfaction Questionnaire.

u WHODAS 2.0: World Health Organization Disability Assessment Schedule, Version 2.0.

v *DSM-5*: *Diagnostic and Statistical Manual of Mental Disorders* (Fifth Edition).

w PRAMS SLE-14: Pregnancy Risk Assessment Monitoring System Stressful Life Events (14-item scale).

x QuickSCID: Quick Structured Clinical Interview for *DSM-5* Disorders.

y TFA: Theoretical Framework of Acceptability: Generic Questionnaire.

z TFA will be completed 1, 3, and 6 weeks after the participant starts their assigned intervention.

#### Primary Clinical Outcome Measures

We use the depression module of the Structured Clinical Interview for *DSM-5* (*Diagnostic and Statistical Manual of Mental Disorders* [Fifth Edition]), Research Version (SCID-5-RV) [21] to assess our primary clinical outcome, incidence of perinatal depression. This binary outcome measures whether a participant experienced a depressive episode at any point since randomization. To reduce recall bias, we conduct interviews at 6 follow-up time points. We assess current and past major depressive episodes with the stem question revised from “Have you ever...” to “Since the last interview on [date], have you ever…”

We use the Edinburgh Postnatal Depression Scale (EPDS) [27] to quantify depressive symptom severity [27]. Participants complete the EPDS at each follow-up time point, allowing us to measure changes in depressive symptoms from baseline to 12 months post partum. The EPDS is a 10-item self-report measure that omits depressive symptoms that can be conflated with normal perinatal symptoms. It is frequently used and validated to assess depressive symptom severity during pregnancy [28,29], with estimates of sensitivity and specificity for detecting major depression ranging from 70%‐100% and 74%‐97%, respectively, depending on cutoff and sample [30]. Among postpartum samples, estimates of sensitivity and specificity are 86% and 78%, respectively [27]. We also use the Patient Health Questionnaire (PHQ-9) to quantify depressive symptom severity [31]. This 9-item self-report measure is frequently used in research and clinical practice.

#### Secondary Clinical Outcome Measures

We assess suicidal ideation and anxiety because perinatal insomnia and depression are associated with suicidal ideation and anxiety [32-34]. We use the *Since Last Contact* version of the Columbia Suicide Severity Rating Scale [35] to assess suicidal ideation, intensity of ideation, and suicidal behavior since the last interview.

We use the Generalized Anxiety Disorder 7-item scale (GAD-7) to assess anxiety symptom severity [36]. The GAD-7 is a 7-item self-report measure with total scores ranging from 0 to 27. Among pregnant and postpartum women, a cutoff of 13 on the GAD-7 has a sensitivity of 61% and a specificity of 73% for detecting diagnoses of generalized anxiety disorder [37].

#### Primary Mediator Measure

We assess insomnia symptom severity using the Insomnia Severity Index (ISI), a 7-item self-report measure that assesses difficulty initiating or maintaining sleep, satisfaction with sleep, impairment, distress, and the extent to which others have noticed symptoms over the last 2 weeks [38]. The ISI had high internal consistency in a clinical sample seeking treatment for insomnia (Cronbach α=0.91) [17]. At screening, we excluded women who were unlikely to meet criteria for insomnia disorder using a cutoff validated for identifying participants in clinical trials (ISI<11) [17]. At baseline, we included those with elevated insomnia symptom severity, defined as ISI≥10 [17], which has been validated for detecting *DSM-5 *insomnia disorder during pregnancy [19].

We also use the *consensus sleep diary* to assess sleep metrics, such as average sleep onset latency, wake after sleep onset, sleep efficiency, and sleep duration. The consensus sleep diary is the gold standard measure of subjective sleep. Research suggests that the consensus sleep diary is associated with subjective and objective measures of sleep, differentiates good sleepers from those with insomnia disorder, and is sensitive to improvements over the course of CBT-I treatment [39].

#### Moderator Measure

We use the EPDS to assess our primary moderator, baseline depressive symptom severity. We selected baseline depressive symptom severity as our primary moderator based on our strong preliminary data, as well as similar findings in a nonperinatal population [14].

### Adverse Events

Adverse events are assessed systematically via interview at each follow-up time point. Participants are asked whether they experienced new or worsening general health problems, emotional problems, or problems with their pregnancy. Interviewers ask open-ended questions to characterize severity, relationship to their assigned condition or study procedures, actions taken, outcome, expectedness, and seriousness. All adverse events are reviewed by a medical monitor.

### Recruitment

Because this is a fully remote trial, we recruited participants through targeted, paid advertisements on Meta platforms (eg, Facebook and Instagram); Everyday Health Group apps and direct emails (What to Expect and BabyCenter); and electronic health record messages to potentially eligible patients. Other recruitment sources included via clinicaltrials.gov, ResearchMatch, social media content creators, and word of mouth.

### Participant Procedures and Timeline

**Figure 1.**
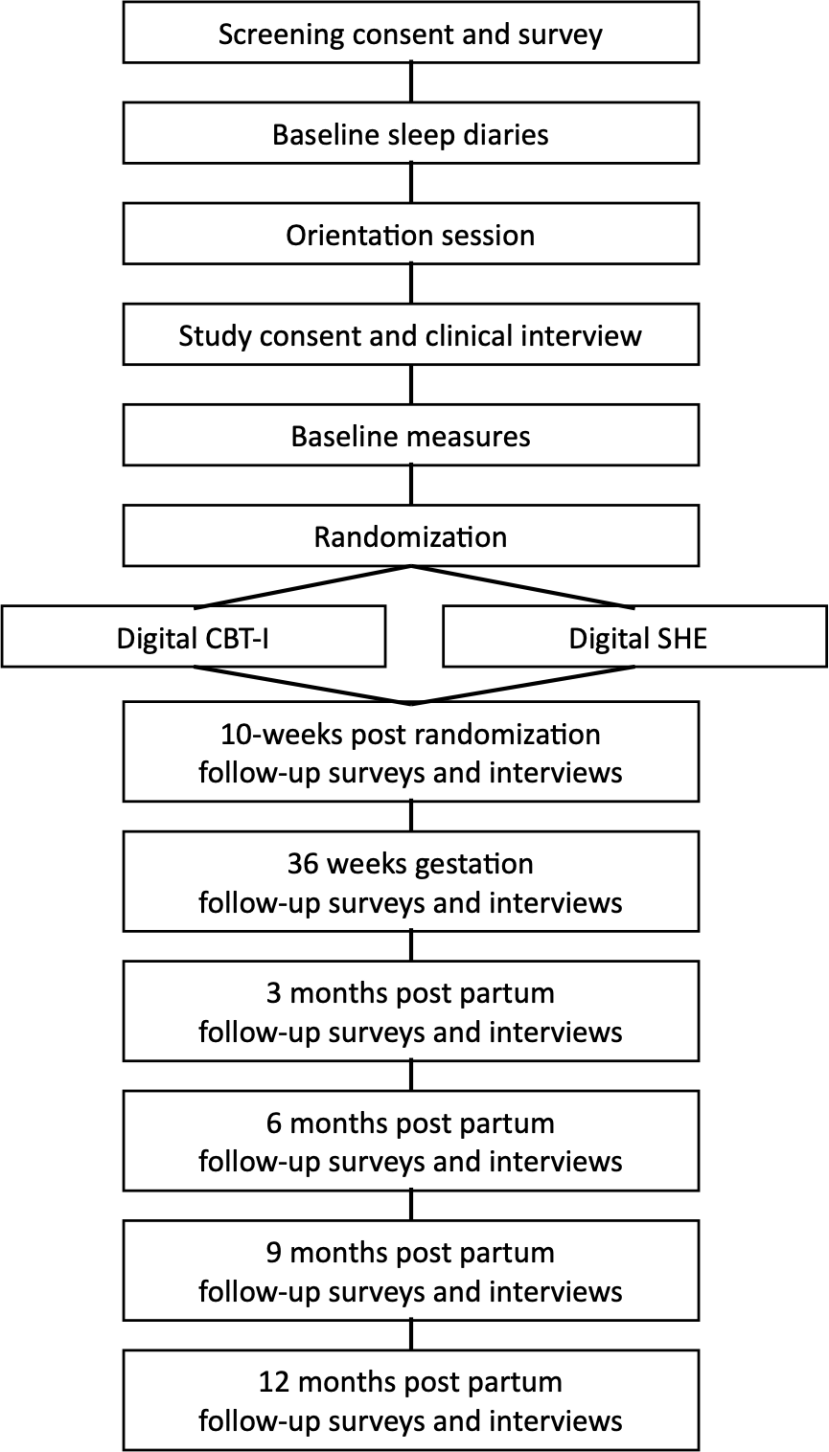
Schematic of study activities.

Figure 1 presents a schematic of study activities.

#### Screening Survey

People interested in participating completed an electronic consent form describing the screening survey, baseline sleep diaries, and orientation session. Those who consented to the screening process completed the online screening survey to determine potential eligibility and provided demographic data. Those who were potentially eligible (eg, ISI≥11) were invited to complete baseline sleep diaries.

#### Baseline Sleep Diaries

Each morning for 7 consecutive days, potential participants received a link to a brief online sleep diary. The sleep diaries served as a behavioral run-in to identify participants who are likely to complete study measures after randomization. Those who completed at least 4 of 7 sleep diaries were invited to complete an orientation session. In addition, for those who were eventually included in the study, the data provided baseline sleep metrics (eg, sleep onset latency and sleep duration).

#### Orientation Session

In a 1-hour orientation session via videoconference, study staff members discussed the importance of the study question, the required commitments, what to expect if randomized to digital CBT-I or digital SHE, the rationale for random assignment, and the detrimental effects of attrition bias on the success of the study.

#### Documentation of Pregnancy

Potential participants were required to provide documentation of their pregnancy, and could opt to do so at the end of the screening survey or after the orientation session. Acceptable forms of documentation included an ultrasound or an after-visit summary with the participant’s name, estimated due date or gestational age, and appointment date. Documentation was reviewed by research coordinators for validity before scheduling the consent and clinical interview. For example, research coordinators confirmed that the participant’s estimated due date and gestational age reported on the screening survey matched the information provided in the pregnancy documentation.

#### Determination of Insomnia Severity

The day before the consenting and clinical interview, participants completed the ISI; those with an ISI total score less than 10 were excluded.

#### Consenting and Clinical Interviews

Study staff administered the revised version of the Structured Clinical Interview for Sleep Disorders overview and insomnia disorder sections. Participants who did not meet the criteria for insomnia disorder were excluded, and the appointment was ended. All others reviewed the study consent form with staff, and those who consented to participate completed the remainder of the clinical interviews. Staff ended the interview if a participant met an exclusion criterion (eg, current major depressive episode). If staff were uncertain about whether a participant met criteria for an excluded disorder, the interview was completed in its entirety, and the segments in question were reviewed by the coauthor (R Manber) before informing the participant of their eligibility.

#### Baseline Measures

Participants who were eligible after completing the clinical interviews completed baseline self-report measures online via REDCap before randomization (Table 2).

#### Randomization and Intervention

Randomization was completed during a telephone call with an eligible participant. At the beginning of the call, staff confirmed the participant’s willingness to complete either intervention and follow-up measures regardless of intervention assignment. After receiving an affirmative response, staff revealed the intervention assignment, informed the participant, and provided information about how to access their intervention. Study staff encouraged the participant to set aside time once a week to complete their intervention sessions. Study staff reviewed the schedule of follow-up measures and instructed the participant not to reveal their intervention assignment to masked follow-up interviewers. Before ending the call, participants completed a brief measure of treatment expectations and credibility. Study staff then emailed all information that was discussed on the call.

#### Follow-Up Measures

At 5 weeks post randomization (ie, halfway to the 10-week postrandomization time point), participants completed brief online measures of insomnia and depressive symptom severity. Subsequently, participants completed follow-up measures via online questionnaires and clinical interviews via videoconferencing at 10 weeks post randomization, 36 weeks of gestation (unless randomized after 24 weeks of gestation or if they gave birth before 35 weeks of gestation), and every 3 months after delivery (ie, 3, 6, 9, and 12 months post partum).

#### Procedures for Elevated Depressive Symptoms and/or Suicidal Ideation

At enrollment, participants were instructed to make immediate contact with study personnel with any questions or concerns regarding worsening depression symptoms or suicidal ideation. Additionally, we provided contact information for therapeutic resources and crisis hotlines.

When completing self-report measures online via REDCap, participants who score ≥10 on the EPDS or PHQ-9 immediately view a pop-up with mental health resources and instructions to call 911 or go to the nearest emergency room if they are in imminent danger of hurting themselves. This information is also sent by email. Email triggers immediately notify study staff members about score elevations. Study staff members subsequently conduct the SCID-5-RV to determine if the participant meets criteria for a major depressive episode. Study staff recommends that any participant who meets criteria for a major depressive episode notify their obstetrician or primary care provider.

Similarly, participants who endorse the suicidality item on the EPDS (item 10 score >0) and/or the PHQ-9 (item 9 score >0) complete the Columbia Suicide Severity Rating Scale self-report survey. Information on suicide and mental health resources is automatically displayed on the same page. Email triggers immediately notify study staff members, who then follow the suicide risk assessment protocol.

### Sample Size

#### Aim 1: Evaluate the Efficacy of Digital CBT-I for Preventing Perinatal Depression

Based on our preliminary data, we expect a 16% lower rate of depression among women in the digital CBT-I condition relative to women in the digital SHE condition. We conservatively power the study to detect a between-group difference of 10% in case the effect is smaller than expected. Standard power calculations for the comparison of 2 proportions show that with α set at .05, chi-square tests with 199 women per group would have power greater than 80% to detect this difference (hypothesis 1a) [40].

The sample size and number of repeated measures per participant are large enough to allow us to detect important differences in the magnitude of within-woman change in depressive symptom severity, measured by the EPDS, between the 2 conditions with sufficient power (hypothesis 1b). We will measure depression outcomes at 7 time points throughout the study period. The statistical analyses will compare within-subject changes in the EPDS over time between the 2 intervention conditions using linear mixed effects models. Sample size calculations for repeated measures studies require estimates of the magnitude of the within-subject correlation of the outcome; data from our previous trial of digital CBT-I provide us with an estimate of 0.5 for this correlation for the EPDS outcome [10]. Our previous trial also provides an estimate of 9.2 units for the within-subject variance of the EPDS measure. Standard power calculations for linear mixed effects models show that with an α set at the .05 level and with 2 conditions of 199 subjects each, we will have power greater than 80% to detect a difference in within-subject slopes of 0.04 (units of EPDS) per month between the 2 intervention groups [41]. In our previous trial, the estimated difference in slopes between digital CBT-I and standard care was 0.12.

#### Aim 2: Test Mediators of the Effect of Digital CBT-I on Perinatal Depression

We base sample size calculations on a statistical test to detect statistically significant mediation of the treatment effect on within-subject change in EPDS through within-subject change in ISI using linear regression. Standard sample size calculations for mediation using linear regression analysis require the specification of the variances of the depression outcome (EPDS), the treatment predictor, and the sleep mediator (ISI), the magnitude of the treatment effects without adjustment for the mediator, and the correlation of the mediator and treatment variables [42]. Data from our previous trial of digital CBT-I provide estimates for all of these quantities. Calculations [42] show that a standard test for mediation using our sample will have power greater than 80% to detect a proportion of treatment effect explained of 0.02. Using data from our previous trial of digital CBT-I for women and changes from baseline to 10 weeks, a linear regression with change in EPDS from baseline to 6 months post partum as the outcome and treatment as the single predictor yielded an estimated regression coefficient of −0.14 (units of EPDS). Adding a change in ISI to the model reduced the estimated treatment effect to −0.12 (units of EPDS), with an estimated proportion of treatment effect of 0.10, far exceeding 0.02.

#### Aim 3: Test Moderation of the Effect of Digital CBT-I on Perinatal Depression

We base sample size calculations on tests of the interaction of treatment condition with depressive symptoms at baseline (using an established cutoff of EPDS <10) in logistic regressions with incidence of probable major depression as the outcome. These sample size methods require the specification of several inputs that describe the anticipated prevalence of the outcome, predictors, and their relationships [43]. Calculations based on inputs from our previous digital CBT-I trial show that the sample of 398 participants (199 per group) provides 80% power to detect an interaction odds ratio (OR; OR for baseline EPDS <10 compared with baseline EPDS ≥10) of 4.1 with binary indicators for condition and depressive symptoms at baseline. Our previous trial yielded an estimated OR of 10, indicating that the detectable interaction OR of 4 is a realistic objective.

These calculations show that the sample size will allow us to detect clinically important differences in depression incidence and changes in depressive symptom severity between women in the 2 intervention conditions, as well as clinically meaningful mediation and moderation effects, with 80% power. Initially, we set our attrition rate at 20% based on our previous trial, yielding a total enrolled sample size of 498. In September 2025, with approval from the Data Safety Monitoring Board (DSMB) and the National Institute of Mental Health, we revised the attrition rate to 10% based on our observed higher-than-anticipated retention, yielding a required sample size of at least 443.

### Randomization

We randomized participants to CBT-I or SHE after the baseline assessment using a 1:1 allocation ratio with blocked randomization to balance the group sizes. A biostatistician not affiliated with the study generated the randomization sequence. Study staff uploaded the masked randomization table to the REDCap database. Once an eligible participant completed baseline measures, unmasked study staff used the randomization module in REDCap to reveal their allocation assignment.

We do not expect to break the randomization code. At their discretion, the DSMB can break the randomization blind to protect participant safety if needed.

### Masking

The principal investigator (JF) and staff members involved in conducting outcome assessments are masked from condition assignment. Although we cannot mask participants from condition assignment, allocation occurred after enrollment to avoid selection bias. We mitigate the effect of participant expectations by comparing 2 credible conditions. At the beginning of follow-up clinical interviews, masked interviewers instruct participants not to reveal their study assignment. If a participant reveals their condition assignment, future interviews are assigned to a different masked staff member.

### Data Collection Methods

Interviewers enter interview data directly into REDCap simultaneous to completing the interview. Interviewers collect as much information as possible to clearly justify their ratings, such that an independent rater who later listens to the recording should have enough information to make a similar rating. Interviewers enter detailed notes on REDCap interview forms. If a participant is unable to complete the entire interview appointment, the interviewer prioritizes collecting data on the primary outcome, incidence of major depression. Interviewers promptly resolve any questions about their ratings with the principal investigator (JF) and coauthor (R Manber).

Participants enter their responses to questionnaires directly into REDCap. Links to the questionnaires are sent via email and SMS text message. Questionnaires have clear instructions and are automatically dated and linked to the participant ID. To minimize missing data, participants are alerted if they miss an item. Where possible, REDCap allows only appropriate and in-range responses to ensure consistency and ease data analysis (eg, a number for a numerical question and a date in the correct format for a date question).

### Data Quality Control

Self-report questionnaires were designed to accept only valid responses. In real time and as close to data collection as possible, study staff members check each submitted self-report measure to identify and correct any potential issues (eg, participant indicated on the sleep diary that she went to sleep at 10 AM instead of 10 PM). Errors and their corrections are documented in a data anomaly log. Clinical research coordinators send reminders about interview appointments and make multiple attempts to contact participants to complete follow-up measures. Similarly, all forms completed by staff (eg, inclusion and exclusion checklists and interview forms) are reviewed to resolve any data quality issues.

### Training and Reliability of Clinical Interviewers

Clinical interviewers are bachelor’s level research coordinators and doctoral-level psychology trainees. Training is led by coauthor (R Manber), and includes intensive asynchronous didactics and experiential training. The didactic training reviewed diagnostic criteria, oriented staff to the interview questions, and discussed challenges for assessing sleep and psychopathology during the perinatal period. Experiential training included rating training videos, shadowing interviews, and practicing interviews with a confederate. Interviews were recorded, and interviewers were required to demonstrate competency before conducting interviews independently. The first 3 independent interviews were reviewed by a coauthor (R Manber). Thereafter, the coauthor (R Manber) is available to provide feedback upon request, when there is diagnostic uncertainty, or when rating discrepancies are identified during quality control. To minimize rater drift, the coauthor (R Manber) leads quarterly reliability meetings to review agreement on diagnostic ratings. At study midpoint and end point, a random subset of 10 interviews per interviewer is selected to assess interrater reliability of the primary outcome data (ie, presence of a major depressive episode).

### Retention

Retaining participants in digital and longitudinal trials is often a significant challenge, and attrition bias can undermine the quality of research. We implement several strategies that we believe contributed to high retention in our previous trials, including using daily sleep diaries as behavioral run-in; conducting an orientation session before enrollment (refer to Participant Procedures and Timeline section for more detail) [44]; creating a recognizable visual project identity (eg, study logo and similar colors and fonts on trial materials); requesting contact information for a backup contact person who can help us locate the participant if we have trouble reaching them; sending birthday cards and study branded baby onesies; and compensating US $50 for completing study procedures at each time point (ie, up to US $350).

Study staff members strive to be gracious. They develop a warm, authentic relationship with participants. Where possible, participants complete follow-up interviews with the same interviewer each time, rather than changing interviewers from time point to time point. Study staff members offer flexible appointment times via a third-party scheduling site (Acuity Scheduling powered by Squarespace), where participants can self-schedule. Participant contacts are responded to within 1 business day. Methods for engaging participants during videoconferencing appointments are detailed elsewhere [45]. Study staff members also strive to be tenacious, including making multiple contact attempts using a range of methods (email, text, and phone call), sending both automated and personalized reminders to complete questionnaires, and contacting a back-up contact, if provided at enrollment. For participants with limited availability for assessments, study staff members prioritize collecting data on the primary outcome measure.

### Statistical Methods

All analyses will be conducted on the intention-to-treat sample, defined as all randomized participants.

For descriptive statistics, categorical data will be presented with frequency and percentage. Continuous data will be presented with mean, SD, median, and range.

Aim 1: To test *hypothesis 1*a, we will compare the rate of major depression among women randomized to digital CBT-I to women randomized to digital SHE using a standard chi-square test for 2 proportions. The outcome is binary and indicates whether a participant experienced a depressive episode at any point since randomization (SCID-5-RV). We will calculate a 95% CI for the difference in the 2 proportions to quantify the magnitude of the CBT-I effect.

To test *hypothesis 1b*, we will compare within-woman changes in depressive symptoms (EPDS, including data from baseline; 10 weeks post randomization; 36 weeks of gestation; and 3, 6, 9, and 12 months post partum) between women in the digital CBT-I and digital SHE groups using linear mixed effects models [46]. These models will include repeated measures of depressive symptoms as the outcome, along with time, treatment group, and time by group interactions as predictors. Models will also include random intercepts to accommodate the correlation among the repeated responses within subjects, and possibly random slopes to accommodate between-subject differences in rates of change. The regression coefficient of the time by group interaction measures the differences in the rate of within-subject change in the outcomes between the treatment groups. We will calculate 95% CIs for the time by group interaction to provide a range of differences in change that are consistent with the data. We will assess the statistical significance of the time by group interaction using likelihood ratio tests. We note that mixed effects models do not require the same number of repeated measures for each subject, so our approach will include all women who provide any data and will be an intention-to-treat approach. We will fit the mixed models using routines in Stata (StataCorp).

Although we expect that randomization will balance baseline characteristics (eg, gestational age, parity, and insomnia symptom severity) between the 2 conditions, we will calculate standardized mean differences to identify any imbalances. If we identify imbalances, we will conduct secondary analyses including imbalanced covariates as predictors. Additionally, we will conduct sensitivity analyses to examine whether findings change substantively when omitting participants with new onset of sleep, medical, or psychiatric disorders, or initiation of mental health treatment, and to examine whether the depression prevention effect of digital CBT-I is dose-dependent by including the number of sessions completed as a covariate.

Aim 2: To test *hypothesis 2*a, we will fit 2 logistic regression models to assess whether change in prenatal insomnia symptoms (ISI) mediates the effect of digital CBT-I on incidence of major depression from baseline to 12 months post partum [47]. The first model will include the binary indicator of depression as the outcome and treatment group as the single predictor. The second model will additionally include subject-specific change in insomnia severity from baseline to 10-weeks post randomization. We will estimate subject-specific changes in insomnia severity as the predicted random slopes from a linear mixed effects model with the repeated measures of insomnia severity as the outcome and time as the single predictor, along with random intercepts and time effects. Differences in the estimated treatment effects between the 2 models indicate mediation by insomnia severity. We will assess whether the conditions for mediation are met by fitting 2 additional models; first, a linear regression model for subject-specific change in insomnia severity on treatment group; and second, a logistic regression model for major depression on subject-specific change in insomnia severity. Statistically significant predictor effects in each of these models are consistent with mediation.

To test *hypothesis 2b*, we will follow a similar approach to assess the potential mediation of the treatment effect on the changes in depressive symptoms (EPDS, including data from baseline; 10 weeks post randomization; 36 weeks of gestation; and 3, 6, 9, and 12 months post partum) by changes in prenatal insomnia severity (ISI) by fitting 2 linear mixed effects regression models. The first model is the linear mixed effects model for repeated measures of depressive symptoms, which we fit to test the hypothesis of aim 1b, namely a model that includes treatment group, time, and the time by group interactions as the predictors, along with random intercepts and time effects. The second model will additionally include repeated measures of insomnia severity (ISI). We will decompose the insomnia severity variable into between- and within-woman components in order to isolate pure within-woman change [48]. Differences in the estimated time by treatment interaction effects between the 2 models indicate mediation by changes in prenatal insomnia severity.

Aim 3: To test *hypothesis 3*a, we will fit a logistic regression model with perinatal depression incidence as the outcome, along with treatment group, baseline depressive symptom severity, and their interaction as the predictors. The regression coefficient of the interaction term measures the difference in the effect of the digital CBT-I intervention as a function of the level of baseline depressive symptom severity. We will test the statistical significance of the interaction using a likelihood ratio test and quantify the magnitude of the interaction effects using 95% CIs for the interaction regression coefficient.

To test *hypothesis 3b*, models will include repeated measures of depressive symptoms as the outcome, along with time, treatment group, and 2-way interactions of depressive symptom severity with time and depressive symptom severity with treatment, as well as a 3-way interaction of time, treatment, and depressive symptom severity. The 3-way interaction term assesses whether the magnitude of the time by treatment group interaction (a primary treatment effect of aim 1) differs as a function of baseline depression. We will assess the statistical significance of the 3-way interaction using a likelihood ratio test and quantify the magnitude of the interaction effects using 95% CIs for the interaction regression coefficient.

### Data Monitoring Committee

A DSMB provides recommendations regarding the continuation, modification, or termination of the PRISM study. The DSMB meets annually to review cumulative data to evaluate safety, study conduct, scientific validity, and data integrity. The DSMB consists of 3 independent members approved by the National Institute of Mental Health with expertise in the fields of obstetrics and gynecology, perinatal sleep and mood, and biostatistics. The DSMB is independent from the sponsor and funder. There are no planned interim analyses. The DSMB reviews any serious adverse events to determine whether the trial should be stopped.

### Ethical Considerations

#### Approval

This study protocol was approved by the Institutional Review Board (IRB) in the Human Research Protection Program at the University of California, San Francisco (UCSF IRB 21‐35440). The Stanford IRB relied on the UCSF IRB as the single IRB of record. Important changes to the original protocol after trial commencement are described in Table 3.

**Table 3.** Important changes to original protocol after trial commencement.

Date	Description of change	Brief rationale
December 28, 2022	Added WHODAS^a^ and *DSM-5*^b^ crosscutting measure.	To comply with NIH^c^ requirements.
February 14, 2023	Operationalized “current elevated insomnia symptom severity” as a baseline ISI^d^ score ≥10.	Eligibility on this criterion was originally defined using a screening ISI score ≥11. Because weeks could elapse between screening and the baseline assessment, we added a baseline severity requirement to ensure insomnia symptoms were present at enrollment.
March 9, 2023	Added PRAMS SLE-14^e^ measure of stressful life events in 12 months before delivery.	May be a potentially important covariate.
April 24, 2023	For baseline assessment of psychotic symptoms, panic disorder, PTSD^f^, using QuickSCID^g^ instead of SCID-5-RV^h^.	To reduce length of time of baseline interviews.
January 12, 2024	Modified allowable response window for 36 weeks’ gestation time point such that surveys and interviews were only collected if >14 days from 10 weeks post randomization time point.	To reduce redundant data collection and participant burden.
January 30, 2024	Added measure to assess treatment feasibility and acceptability (TFA^i^).	To address aims of diversity supplement.
April 3, 2024	Clarified eligibility criteria to indicate that participants taking medications with antidepressant effects will be excluded (eg, Lamictal), even if not technically an “antidepressant medication.”	To align with the original intent of this criterion to exclude those for whom the antidepressant effects of the intervention would be difficult to interpret.
January 30, 2025	Clarified eligibility criteria to indicate that participants receiving antidepressant treatments (eg, TMS^j^) will be excluded, even if not a “medication.”	To align with the original intent of this criterion to exclude those for whom the antidepressant effects of the intervention would be difficult to interpret.
September 23, 2025	Updated sample size estimate.	Initially, we set our attrition rate at 20% based on our previous trial, yielding a total enrolled sample size of 498. With approval from the DSMB^k^ and NIMH^l^, we revised the attrition rate to 10% based on our observed higher than anticipated retention, yielding a required sample size of at least 443.

a WHODAS: World Health Organization Disability Assessment Schedule.

b *DSM-5*: *Diagnostic and Statistical Manual of Mental Disorders* (Fifth Edition).

c NIH: National Institutes of Health.

d ISI: Insomnia Severity Index.

e PRAMS SLE-14: Pregnancy Risk Assessment Monitoring System Stressful Life Events (14-item scale).

f PTSD: posttraumatic stress disorder.

g QuickSCID: Quick Structured Clinical Interview for *DSM-5* Disorders.

h SCID-5-RV: Structured Clinical Interview for *DSM-5* Disorders, Research Version.

i TFA: Theoretical Framework of Acceptability.

j TMS: transcranial magnetic stimulation.

k DSMB: Data and Safety Monitoring Board.

l NIMH: National Institute of Mental Health.

#### Consent

Because this study involves no in-person visits, all consent occurred electronically. There were 3 consent forms—the screening consent, the future contact consent, and the study consent. Consent forms were written at an 8th-grade education level, used lay language, and provided information about the background and purpose of the study, scope and length of participation, study procedures, legal and ethical limits to confidentiality, clinical interview audio and videotaping procedures, risks, benefits, alternatives, ability to discontinue participation, privacy and confidentiality, compensation, and whom to contact with questions. Potential participants were assured that their decision to participate or decline participation in the study would have no effect on their current or future receipt of health care services at UCSF or affiliated clinics.

Screening consent: First, potential participants viewed an introduction page that briefly described the study. Next, the screening consent form described the enrollment procedures (ie, screening survey, daily sleep questionnaires, orientation appointment, consent, and clinical interview). Participants who declined to participate were automatically directed to the end of the survey and thanked for their time. Participants who agreed to participate then began the screening survey.

Future contact consent: After completing the screening survey, regardless of eligibility, participants were given the opportunity to provide their contact information to be contacted for future research opportunities.

Study consent: The orientation session provided another opportunity to engage participants in the informed consent process (refer to Participant Procedures and Timeline section for more details). Individuals who were interested in participating after the orientation session were scheduled for an individual appointment to complete the consent process and the clinical interview to determine eligibility. Consenting participants provided an electronic signature via REDCap and received a signed copy of their consent form via email.

#### Confidentiality

All participant data were collected and stored in REDCap, a secure, HIPAA (Health Insurance Portability and Accountability Act)-compliant platform with role-based access controls, encrypted data transmission, isolated identifier storage, and full audit trails.

## Results

Participant recruitment is complete. The first participant was randomized in November 2022, and the final participant was randomized in June 2025. A total of 456 participants were randomized, exceeding our target of 443. We expect to complete data collection in December 2026.

## Discussion

Prenatal insomnia is a robust risk factor for perinatal depression, but it is unknown whether treating prenatal insomnia prevents perinatal depression. Our preliminary data are encouraging, and we are now conducting a confirmatory efficacy trial that is sufficiently powered and designed to provide definitive evidence. If successful, we will have a new, scalable way to prevent perinatal depression and avert its wide-reaching consequences. This research has high public health significance because perinatal depression is common and consequential, there are few intervention options for preventing perinatal depression, no prevention programs target prenatal insomnia, and the accessibility of existing perinatal depression prevention interventions is currently limited [49].

## Supplementary material

10.2196/90969 Checklist 1 SPIRIT v2025 checklist.

## Abbreviations

CBT-I: cognitive behavioral therapy for insomnia
*DSM-5*: *Diagnostic and Statistical Manual of Mental Disorders* (Fifth Edition)
DSMB: Data and Safety Monitoring Board
EPDS: Edinburgh Postnatal Depression Scale
GAD-7: Generalized Anxiety Disorder 7-item scale
HIPAA: Health Insurance Portability and Accountability Act
IRB: Institutional Review Board
ISI: Insomnia Severity Index
OR: odds ratio
PHQ-9: Patient Health Questionnaire
PRISM: Perinatal Research for Improving Sleep and Mental Health
RCT: randomized controlled trial
REDCap: Research Electronic Data Capture
SCID-5-RV: Structured Clinical Interview for *DSM-5* Disorders, Research Version
SHE: sleep hygiene education
SPIRIT: Standard Protocol Items: Recommendations for Interventional Trials
UCSF: University of California, San Francisco

## References

[1] Gavin NI, Gaynes BN, Lohr KN, Meltzer-Brody S, Gartlehner G, Swinson T (2005). Perinatal depression: a systematic review of prevalence and incidence. Obstet Gynecol.

[2] Khalifeh H, Hunt IM, Appleby L, Howard LM (2016). Suicide in perinatal and non-perinatal women in contact with psychiatric services: 15 year findings from a UK national inquiry. Lancet Psychiatry.

[3] Stein A, Pearson RM, Goodman SH (2014). Effects of perinatal mental disorders on the fetus and child. Lancet.

[4] Luca DL, Garlow N, Staatz C, Margiotta C, Zivin K (2019). Societal costs of untreated perinatal mood and anxiety disorders in the United States. https://policycentermmh.org/app/uploads/2024/05/IBNationalSocietalCostsUntreatedPMAD.pdf.

[5] O’Connor E, Senger CA, Henninger ML, Coppola E, Gaynes BN (2019). Interventions to prevent perinatal depression: evidence report and systematic review for the US Preventive Services Task Force. JAMA.

[6] Baglioni C, Battagliese G, Feige B (2011). Insomnia as a predictor of depression: a meta-analytic evaluation of longitudinal epidemiological studies. J Affect Disord.

[7] Felder JN, Laraia B, Coleman-Phox K (2018). Poor sleep quality, psychological distress, and the buffering effect of mindfulness training during pregnancy. Behav Sleep Med.

[8] Tomfohr LM, Buliga E, Letourneau NL, Campbell TS, Giesbrecht GF (2015). Trajectories of sleep quality and associations with mood during the perinatal period. Sleep.

[9] Suri R, Stowe ZN, Cohen LS (2017). Prospective longitudinal study of predictors of postpartum-onset depression in women with a history of major depressive disorder. J Clin Psychiatry.

[10] Felder JN, Epel ES, Neuhaus J, Krystal AD, Prather AA (2020). Efficacy of digital cognitive behavioral therapy for the treatment of insomnia symptoms among pregnant women: a randomized clinical trial. JAMA Psychiatry.

[11] Manber R, Bei B, Simpson N (2019). Cognitive behavioral therapy for prenatal insomnia: a randomized controlled trial. Obstet Gynecol.

[12] Kalmbach DA, Cheng P, O’Brien LM (2020). A randomized controlled trial of digital cognitive behavioral therapy for insomnia in pregnant women. Sleep Med.

[13] Felder JN, Epel ES, Neuhaus J, Krystal AD, Prather AA (2022). Randomized controlled trial of digital cognitive behavior therapy for prenatal insomnia symptoms: effects on postpartum insomnia and mental health. Sleep.

[14] Cheng P, Kalmbach DA, Tallent G, Joseph CL, Espie CA, Drake CL (2019). Depression prevention via digital cognitive behavioral therapy for insomnia: a randomized controlled trial. Sleep.

[15] Prendeville W, Grudzinskas JG, O’Brien PMS (1998). Problems in Early Pregnancy: Advances in Diagnosis and Management.

[16] Anderson M (2019). Mobile technology and home broadband 2019. https://www.pewresearch.org/internet/wp-content/uploads/sites/9/2019/06/PI_2019.06.13_Mobile-Technology-and-Home-Broadband_FINAL2.pdf.

[17] Morin CM, Belleville G, Bélanger L, Ivers H (2011). The Insomnia Severity Index: psychometric indicators to detect insomnia cases and evaluate treatment response. Sleep.

[18] Qaseem A, Kansagara D, Forciea MA, Cooke M, Denberg TD, Clinical Guidelines Committee of the American College of Physicians (2016). Management of chronic insomnia disorder in adults: a clinical practice guideline from the American College of Physicians. Ann Intern Med.

[19] Kalmbach DA, Cheng P, Roth A (2022). DSM-5 insomnia disorder in pregnancy: associations with depression, suicidal ideation, and cognitive and somatic arousal, and identifying clinical cutoffs for detection. Sleep Adv.

[20] Taylor DJ, Wilkerson AK, Pruiksma KE (2018). Reliability of the structured clinical interview for DSM-5 sleep disorders module. J Clin Sleep Med.

[21] First MB, JbW W, Karg RS, Spitzer RL (2015). Structured Clinical Interview for DSM-5—Research Version (SCID-5 for DSM-5, Research Version; SCID-5-RV).

[22] Facco FL, Ouyang DW, Zee PC, Grobman WA (2012). Development of a pregnancy-specific screening tool for sleep apnea. J Clin Sleep Med.

[23] Moss TG, Lachowski AM, Carney CE (2013). What all treatment providers should know about sleep hygiene recommendations. Behav Ther (N Y N Y).

[24] Felder JN, Hartman AR, Epel ES, Prather AA (2020). Pregnant patient perceptions of provider detection and treatment of insomnia. Behav Sleep Med.

[25] Carney CE, Edinger JD, Kuchibhatla M (2017). Cognitive behavioral insomnia therapy for those with insomnia and depression: a randomized controlled clinical trial. Sleep.

[26] Chan AW, Boutron I, Hopewell S (2025). SPIRIT 2025 statement: updated guideline for protocols of randomized trials. JAMA.

[27] Cox JL, Holden JM, Sagovsky R (1987). Detection of postnatal depression. Development of the 10-item Edinburgh Postnatal Depression Scale. Br J Psychiatry.

[28] Matthey S, Henshaw C, Elliott S, Barnett B (2006). Variability in use of cut-off scores and formats on the Edinburgh Postnatal Depression Scale: implications for clinical and research practice. Arch Womens Ment Health.

[29] Murray D, Cox JL (1990). Screening for depression during pregnancy with the Edinburgh Depression Scale (EDDS). J Reprod Infant Psychol.

[30] Kozinszky Z, Dudas RB (2015). Validation studies of the Edinburgh Postnatal Depression Scale for the antenatal period. J Affect Disord.

[31] Kroenke K, Spitzer RL, Williams JB (2001). The PHQ-9: validity of a brief depression severity measure. J Gen Intern Med.

[32] Gelaye B, Addae G, Neway B (2017). Poor sleep quality, antepartum depression and suicidal ideation among pregnant women. J Affect Disord.

[33] Osnes RS, Roaldset JO, Follestad T, Eberhard-Gran M (2019). Insomnia late in pregnancy is associated with perinatal anxiety: a longitudinal cohort study. J Affect Disord.

[34] Furtado M, Van Lieshout RJ, Van Ameringen M, Green SM, Frey BN (2019). Biological and psychosocial predictors of anxiety worsening in the postpartum period: a longitudinal study. J Affect Disord.

[35] Posner K, Brown GK, Stanley B (2011). The Columbia-Suicide Severity Rating Scale: initial validity and internal consistency findings from three multisite studies with adolescents and adults. Am J Psychiatry.

[36] Spitzer RL, Kroenke K, Williams JBW, Löwe B (2006). A brief measure for assessing generalized anxiety disorder: the GAD-7. Arch Intern Med.

[37] Simpson W, Glazer M, Michalski N, Steiner M, Frey BN (2014). Comparative efficacy of the generalized anxiety disorder 7-item scale and the Edinburgh Postnatal Depression Scale as screening tools for generalized anxiety disorder in pregnancy and the postpartum period. Can J Psychiatry.

[38] Bastien CH, Vallières A, Morin CM (2001). Validation of the Insomnia Severity Index as an outcome measure for insomnia research. Sleep Med.

[39] Maich KHG, Lachowski AM, Carney CE (2018). Psychometric properties of the consensus sleep diary in those with insomnia disorder. Behav Sleep Med.

[40] Cohen J (1988). Statistical Power Analysis for the Behavior Sciences.

[41] Diggle PJ, Heagerty PJ, Liang KY, Zeger SL (2002). Analysis of Longitudinal Data.

[42] Vittinghoff E, Sen S, McCulloch CE (2009). Sample size calculations for evaluating mediation. Stat Med.

[43] Demidenko E (2008). Sample size and optimal design for logistic regression with binary interaction. Stat Med.

[44] Goldberg JH, Kiernan M (2005). Innovative techniques to address retention in a behavioral weight-loss trial. Health Educ Res.

[45] McClelland B, Ponting C, Levy C (2024). Viewpoint: challenges and strategies for engaging participants in videoconferencing appointments. Contemp Clin Trials.

[46] McCulloch CE, Models M, Neuhaus JM (2008). Generalized, Linear, and Mixed Models.

[47] Vittinghoff E, Glidden DV, Shiboski SC, McCulloch CE (2012). Regression Models in Biostatistics.

[48] Neuhaus JM, McCulloch CE (2006). Separating between- and within-cluster covariate effects by using conditional and partitioning methods. J R Stat Soc Series B Stat Methodol.

[49] Felder JN (2019). Implementing the USPSTF recommendations on prevention of perinatal depression-opportunities and challenges. JAMA Intern Med.

